# Competency in supportive supervision: a study of public sector medicines management supervisors in Uganda

**DOI:** 10.1186/s40545-017-0121-y

**Published:** 2017-10-11

**Authors:** Rachael Henry, Lynda Nantongo, Anita Katharina Wagner, Martha Embrey, Birna Trap

**Affiliations:** 1 Management Sciences for Health, Plot 15, Princess Anne Drive, Bugolobi, P.O. Box 71419, Kampala, Uganda; 2 Harvard Pilgrim Health Care Institute, 133 Brookline Avenue, 6th Floor, Boston, MA 02215 USA; 3 Management Sciences for Health, 4301 N. Fairfax Drive, Suite 400, Arlington, VA 22203 USA; 4 USAID/Uganda Health Supply Chain Program, Management Sciences for Health, Plot 15, Princess Anne Drive, Bugolobi, P.O. Box 71419, Kampala, Uganda

**Keywords:** Supervision, Supportive supervision, Medicines, Medicines management, Uganda

## Abstract

**Background:**

Supportive supervision has been found to be more effective than corrective fault-oriented inspections. Uganda’s Ministry of Health in 2012 implemented a comprehensive strategy (SPARS) to build medicines management capacity in public sector health facilities. The approach includes supportive supervision. This structured observational study assesses supportive supervision competency among medicines management supervisors (MMS).

**Method:**

The study used structured observations of two groups of five purposely selected MMS—one group supervising facilities with greater medicines management improvement during one year of SPARS and one group with less improvement, based on quantitative metrics. We observed and scored behaviors and skills of supervisors in 11 categories deemed critical for effective and supportive supervision.

**Results:**

Supportive supervision was not evenly or adequately implemented, with the median supportive supervision competency score for all observed supervisors being 38%. Supervisors’ main strengths were *problem identification, data interpretation, education,* and *providing constructive feedback* (45%–47%). Their weakest areas were a*ssuring continuity* and *setting targets* (17%), and most MMS were fair to strong in *effective communication, use of tools,* and *problem solving.* MMS of facilities with little improvement in medicines management over time were weak in *setting targets* and *promoting participation*. There was a 33 percentage point difference in the median supportive supervision competency scores between MMS of facilities with more versus less improvement (57%–24%) and a 77 percentage point difference in competency between the highest and lowest scoring MMS (77%–0%). We did not find a relationship between MMS experience (number of visits implemented) and their supportive supervision competency or facility improvement in medicines management. However, there is a likely relationship between supportive supervision competency and facility improvement.

**Conclusion:**

Competency of MMS in supportive supervision among the sampled MMS was generally weak, but with much individual variation. Our results suggest that MMS’ supportive supervision competency is positively related to the SPARS effectiveness scores of the facilities they supervise. We recommend strategies to strengthen supportive supervision behaviors and skills.

**Electronic supplementary material:**

The online version of this article (10.1186/s40545-017-0121-y) contains supplementary material, which is available to authorized users.

## Background

The shortage of pharmacists and health professionals trained in pharmaceutical management in Uganda is well documented [1]. For every 100,000 citizens, there are only 1.6 pharmacists; only 8% of public sector pharmacist posts and 61% of pharmacy technician posts were filled in 2013/14 [2]. Because of this shortage, all categories of health workers are involved at some point in managing medicines, including nurses, midwives, laboratory technicians, clinical officers, doctors, and even social workers. Most of these health workers have insufficient training in keeping track of stock and dispensing. Surveys of public sector facilities depict a challenging environment; in 2010, less than 10% of facilities had all six vital tracer medicines available, no facilities filled in stock cards correctly, and only 1% of facilities provided the correct treatment for a simple cough and cold [1, 3]. It was against this background that the Ministry of Health in 2012 introduced a new national capacity building strategy—SPARS. SPARS combines *supervision* in the form of “supportive supervision” with *performance assessment*, to track improvement and identify problem areas to guide supervision, and a *recognition strategy* to reward good performance.

SPARS’ medicines management supervisors (MMS) visit health facilities to build health workers’ capacity to improve the availability, supply chain management, and use of essential medicines and health supplies, which are essential to health system performance [4]. The MMS assess facility performance in medicines management at each visit using a standard tool with 25 indicators covering five areas: stock management, store management, ordering and reporting, prescribing, and dispensing. The MMS identify areas needing improvement, and they address these areas with on-the-job training, joint problem solving, and target setting. Supervisor feedback, targets, and performance scores are recorded in a supervision book that remains at the facility for staff to act on and for follow-up at subsequent visits.

Several studies in other countries have highlighted the importance of applying supportive supervision (SS) rather than more corrective fault-oriented supervision to improve service delivery [5–7]. SS focuses on the supervisor as a mentor and the supervisee’s participation through open discussion. The supervisees should feel that they have a valued voice that shapes decisions [6]. Although SS is a powerful approach to building professional capacity, studies have shown that the approach and manner in which SS is implemented affects the intervention outcome [6, 8–10].

SPARS is proving effective—21% of 1300 facilities supervised for 12 months reached an acceptable SPARS score of 18.8 (75% of the maximum SPARS score of 25), but improvement varied considerably between facilities [11]. The objectives of our observational study was to assess supportive supervision competency among MMS implementing the SPARS intervention.

## Methods

### Design

Our study used structured quantitative observation to assess MMS’ competency in providing SS. A team of two trained data collectors observed MMS during one facility visit and scored their behavior using a structured checklist and scoring sheet.

### Sampling

MMS selection was done based on previously observed medicines management performance change within the supervised facilities. We used the *SPARS effectiveness scores (*SPARS-ES) assessed for the facilities the MMS had supervised to classify them and compare their measures of competency. *SPARS-ES*, which was measured as the average change in the SPARS score (on the 25-point SPARS scale) between two SPARS visits, including up to five visits per facility from May 2011 to March 2013 and averaging across the facilities supervised by the MMS. SPARS data were extracted from Uganda’s pharmaceutical information portal into which all SPARS performance assessment scores are entered after each facility visit. We ranked all 213 MMS active in the SPARS program by their calculated SPARS-ES score from the highest score (4.00) to the lowest score (0.27). Using the cutoff SPARS-ES score of 1.5., we divided the MMS into two groups-an effective (SPARS-ES >1.5) and a less effective (SPARS-ES ≤ 1.5) group with about 50% of the MMS in each group.

From each of the two SPARS-ES groups, we randomly selected 15 MMS and from each group of 15 MMS we purposely selected five MMS for a total of ten MMS for inclusion in the study. The purposeful selection of the ten MMS was done to ensure optimal diversity in the small sample with respect to professional backgrounds (clinical or pharmaceutical training), geographic representation and inclusion of both district MMS and health sub-district MMS (Table 1). All MMS were male and none were stationed at the facility where they worked. One MMS was excluded due to illness and replaced with an MMS with a similar SPARS-ES from the same district. The facilities supervised by MMS with high SPARS performance scores had a median SPARS-ES of 3.7 per visit. Facilities supervised by MMS with low SPARS performance had a median SPARS-ES of 1.1 per visit. Facilities supervised by MMS with high and with low SPARS-ES scores had similar SPARS baseline scores (see results section below).

**Table 1:** Characteristics of selected MMS

Region	Districts	Facility Hosp./PHC^a^	MMS District Job
Central	Luwero	Hospital	Pharmacy Technicians (P)^b^
		PHC	Clinical Officer (C)^c^
	Jinja	PHC	Clinical Officer (C)
North	Apac	PHC	Clinical Officer (C)
	Oyam	PHC	Nurse In-charge (C)
		PHC	Clinical Officer (C)
West	Kasese	Hospital	District Assistant Drug Inspector (P)
	Kabarole	Hospital	Clinical Officer (C)
East	Kumi	PHC	Pharmacy Technicians (P)
	Mayuge	PHC	District Assistant Drug Inspector (P)

^a^ *PHC* Primary health care facility

^b^ *P* Pharmacy trained

^c^ *C* Clinician

We chose three hospitals and seven primary health care facilities (nine public sector and one non-for profit) to observe MMS based on their pre-planned supervision schedule and convenience.

### Data collection

A researcher (RH) and a pharmacist experienced in SPARS implementation (LN) constituted the observation and data collection team. The researcher observed and rated all ten MMS and only when needed did the pharmacists provide input or technical insight to the observation. The health facility was given prior notice to ensure staff availability for the study visit, and the pharmacist introduced the team to the facility and explained their purpose as introducing a new person (RH) to learn about SPARS supervision. The pharmacist having a strong SPARS background, could also respond to any technical questions that arose during the visit. The research team interviewed ten MMS, ten supervisees, eight in-charges and eight DHOs between October and November 2013, using a structured questionnaire to provide context to the findings from the observation of behaviors and to highlight aspects that could help to improve SPARS visits in the future (Additional file 1). Interview responses are not included in the Results section but are included in the Discussion to provide context, for the observed SS skills and behaviors. All tools were piloted at one SPARS supervisory visit performed by an MMS not included in the study, after which the tools were updated. We excluded the pilot finding from the analysis.

### SS competency assessment

To measure SS competency, we defined 11 behavior and skill categories deemed critical for effective SS. These behaviors and skills had been emphasized in MMS training and in previous research [5, 6]. To reduce assessment subjectivity and ambiguity, we developed an observation indicator checklist based on what the MMS should do or say to show that they had mastered the behaviors and skills in the 11 categories (Table 2). The observation team was blinded to the MMS facilities’ SPARS-ES to avoid bias. Given prior research [5, 6] and the intent of SS, the behavior rating criteria have face validity.

**Table 2:** Supportive supervision behavior and skill categories and linked indicators

Behavior/skill category and definition	Observation indicator checklist
Establishes purpose – Communicates the purpose and aims of SPARS and establishes it as ‘supportive supervision’.	✓ Outlines SPARS and its objectives ✓ Outlines the objectives of the visit ✓ Involves the in-charge ✓ Explains the MMS are not here to police but work together as a team ✓ Relates supervision to the bigger picture - patient/ health system/availability ✓ Explains how SPARS will make their work more efficient in the long-term
Identifies problems – Empowers staff to identify problems as areas that need improvement, not as criticism. Discusses causes and has the facility take ownership of the problem.	✓ Follows up on problems identified at previous visit ✓ Asks staff if they are experiencing any problems ✓ Encourages deeper discussion - about the cause or solution to a problem’. ✓ Encourages staff to identify facility problems ✓ Uses performance assessment to identify problems ✓ Prioritizes by focusing on the most important issue
Communicates effectively – Establishes rapport and acts with a considerate, sympathetic, open, and approachable manner. Is confident and competent, not arrogant or authoritative and establishes a relationship with supervisees so they look forward to and not dread MMS visits.	✓ Greets people by name ✓ Voice audible ✓ Jokes/lighthearted conversation ✓ Creates a relaxed atmosphere ✓ Clear and easy to understand ✓ Sensitive and sympathetic ✓ Good eye contact ✓ Not rude, sarcastic, or arrogant ✓ Confident/professional
Promotes participation – Asks questions, promotes discussion, and listens. Assures that supervisees feel their opinions influence the outcome. They should own the program and not feel it is imposed from above.	✓ Acts like one of the team, not an outsider ✓ Uses ‘we’ to discuss issues ✓ Gets supervisees to ask questions ✓ Asks supervisees opinion ✓ Listens ✓ Promotes discussion ✓ Seeks consensus rather than instructing ✓ Makes supervisees feel comfortable and not tense
Interprets data – Collects accurate data and correctly interprets data to assess performance. Uses performance assessment to motivate staff.	✓ Collects data for performance assessment ✓ Explains data collection and how it relates to performance ✓ Links data to performance, to quality of care and benefit to the community ✓ Collects reliable data ✓ Understands how to interpret data ✓ Uses data to identify gaps/ problems ✓ Encourages staff to interpret and discuss data
Solves problems – Promotes discussion and consensus so the facility owns the solution and is committed to implementing changes. The solution should be detailed and realistic.	✓ Discusses the cause of the problem ✓ Involves supervisee - asks about their suggestions ✓ Provides accurate and constructive advice ✓ Suggests realistic solutions ✓ Makes innovative and creative suggestions ✓ Involves relevant people required to ensure the problem can be solved ✓ Proactive—offers to follow up with decision makers
Uses tools – Uses SPARS tools and practices appropriately to ensure an objective and reliable supervision process that motivates supervisees and helps them understand priorities and expectations. Uses SPARS data collection tool, fills in the supervision book, sets targets, and fills out the spider graph to illustrate performance and achievements.	✓ Uses the data collection tool with SPARS indicators ✓ Leaves a copy of the data collection tool at the facility ✓ Displays the spider graph at the facility ✓ Seeks supervisees views ✓ Writes feedback in the supervision book ✓ Writes targets in the supervision book ✓ Gets the in-charge to sign the supervision book ✓ Updates the spider graph ✓ Uses the performance assessment as a tool to motivate supervisees
Sets targets – Jointly prioritizes realistic targets and makes implementation plan to address identified problem areas to ensure genuine commitment to improvement. Records targets in the supervisory book and follows up at next visit.	✓ Talks about targets agreed at the last visit ✓ Sets targets to complete by next visit ✓ Targets are specific, measurable, accurate, time bounds and realistic ✓ Agrees on targets with staff ✓ Sets timeframe to complete ✓ Sets targets based on performance assessment weaknesses ✓ Gives them tools, strategies, advice, or innovations
Educates –Allows adequate time to educate and instruct supervisee. Demonstrates, uses examples, and provides reasons to ensure the supervisee understands and can implement changes on his or her own. Is patient, clear, and persistent.	✓ Prioritizes time to build capacity (doesn’t just collect data) ✓ Identifies gaps in skills and knowledge ✓ Gives training when it’s needed ✓ Physically demonstrates a task ✓ Gives full explanations ✓ Gives concrete, relevant examples ✓ Ensures supervisee understands, have them demonstrate their understanding ✓ Patiently repeats if they do not understand ✓ Provides correct information and training
Provides constructive feedback – Conducts a formal feedback session on performance and targets with facility staff, supervisee, and In-charge at the end of the visit. Provides reasons not rules and encourages discussion to promote ownership.	✓ Gives feedback based on evidence from the performance assessment ✓ Involves appropriate staff and in-charge in feedback if available ✓ Praises where appropriate ✓ Provides diplomatic, tactful, and constructive correction where appropriate ✓ Does not only finds fault ✓ Gives reasons rather than rules ✓ Motivates—provides incentives for change ✓ Doesn’t overwhelm
Assures continuity – Focuses on continuity to ensure ideas for improvement are not lost. Is mindful of facilities’ priorities and workload when scheduling. Follows up on the previous visit and sets dates for the next visit.	✓ Does not arrive unannounced ✓ Discusses previous visit ✓ Sets a date for the next visit ✓ Discusses what needs to be done before the next visit ✓ Has staff confirm what they will do before the next visit ✓ Gives an idea what the objectives will be at the next visit

### Data analysis

We used MS Excel to calculate the SS overall and category competency scores based on the observation checklist results for each MMS. Each of the 11 SS competency categories had a maximum score of “5” based on six to nine indicators, resulting in an overall maximum score of 55. For each indicator, yes was scored as “1” and no as “0” and the category score calculated proportionally, so that each category had a possible maximum score of five (i.e., total number of yes scores divided by the total number of reported indicators in the category multiplied by five). This adjustment allowed us to compare across the 11 categories. The overall SS competency score for each MMS was calculated by adding the scores of all 11 SS category competency scores against the maximum score of 55 as a percentage. We present summary and category competency scores for all 10 MMS and describe those scores for the five MMS supervising facilities with higher SPARS-ES and the five MMS supervising facilities with lower SPARS-ES scores. We analyzed the SPARS score at the baseline visits for the 10 MMS (43 baseline visits conducted by the MMS of the lower SPARS-ES facilities and 44 by MMS with the higher SPARS-ES) to ensure they were comparable at baseline using simple t-test with equal variances. Given the small sample, we did not perform statistical analyses on the competency ratings.

### Ethical considerations

This study assesses supportive supervision implemented as part of the national capacity-building strategy carried out by MMS under the Ministry of Health. The study did not involve patients, human or personal data, human tissue, or animals. Therefore, the study did not require ethical approval or a waiver. All observations and interviews were conducted with the permission of Ministry of Health, the DHO, the facility in-charges, and the MMS, and all information was kept anonymous.

## Results

### Supportive supervision competency by facility SPARS-ES

For each MMS, the overall SS competency scores, the number of visits implemented in 2012, and their facilities’ SPARS-ES are listed in Table 3. The overall median SPARS-ES was 2.0, ranging from 1.1 to 3.7 points of SPARS score improvement per visit. The median SS competency score for all MMS was 38%, with 33 percentage points difference in the quality of supportive supervision between MMS of facilities with high and those with low SPARS-ES and with 77 percentage points (from 0% to 77%) difference between the lowest and highest scoring MMS observed.

**Table 3:** Summary of facility SPARS effectiveness scores (SPARS-ES), MMS supportive supervision (SS) competency scores and MMS experience*

Medicines management supervisors (MMS)	SPARS –ES**	Supportive supervision competency score (%) median	Total # of SPARS visits implemented in 2012*
MMS of facilities with high SPARS-ES			
MMS a (C)	4.0	56%	20
MMS b (P)	4.0	0%	6
MMS c (C)	3.7	71%	25
MMS d (C)	2.3	40%	43
MMS e (C)	1.6	77%	11
Median	3.7	57%	20
MMS of facilities with low SPARS-ES			
MMS f (P)	1.4	22%	8
MMS g (P)	1.4	37%	24
MMS h (C)	1.1	22%	14
MMS i (P)	0.5	17%	19
MMS j (C)	0.3	55%	22
Median	1.1	24%	19
Median overall	2.0	38%	19

*MMS experience defined as total number of SPARS visits implemented 2012

^**^average facility improvement in medicines management across visits by the MMS in 2012, on a scale from 0 to 25; C: clinician; P: pharmacy-trained

### Supportive supervision competency scores by SS behavior and skills categories

**Fig. 1:**
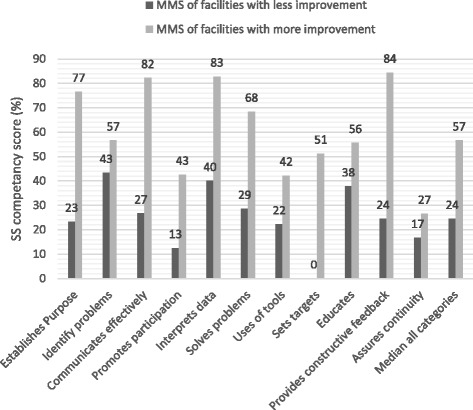
Median SS competency scores by skill and behavior categories among MMS of facilities with high and low SPARS-ES (*n* = 10)

Figure 1 summarizes the SS competency scores on each of the 11 categories for MMS categorized by SPARS-ES of the facilities the MMS supervised. The overall median competency score on all 11 behavior/skill categories and across all MMS was 38%. Median SS competency scores of the five MMS who supervised facilities with higher SPARS-ES were higher (57%) than scores of MMS who supervised lower SPARS-ES facilities (24%).

In the 11 individual behavior and skill categories, there was a marked difference between the two MMS groups; the median differences ranged from 50 to 60% points in the categories *gives constructive feedback, communicates effectively,* and *establishes purpose and sets targets*. SS competency scores of MMS supervising higher and lower SPARS-ES facilities were similar in the categories *identifies problems, educates*, *uses tools,* and *assures continuity,* with differences of less than 20% points. *Use of tools* and *problem identification* were heavily emphasized during the MMS training and are needed to carry out the performance assessment part of the SPARS process for which the MMS receive expense reimbursement. *Assuring continuity* was weakly implemented by both groups. In all SS categories, the group of five MMS supervising facilities with higher SPARS-ES had higher median SS competency scores than those supervising lower SPARS-ES facilities.

We found that the six clinically trained MMS had higher SS competency compared to the four pharmacy/stores management trained MMS (median SS competency score 54.5% versus 18.5%, respectively).

The median number of SPARS visits in year 2012 was similar for both groups—MMS of facilities with higher SPARS-ES completed 20 visits (range 6–43) compared to MMS of facilities with lower SPARS-ES who completed 19 visits (range 8–24). The total number of visits implemented in 2012 by an MMS was used as a measure of experience and evaluated to see if there was a possible relationship between experience, SPARS-ES, and SS competency score. Though we found a difference in SS competency scores between MMS of high and low SPARS-ES facilities (median 57; range 0%–77% compared to median 24; range 17%–55% respectively), both groups performed almost the same number of supervisory visits having similar levels of experience.

We also evaluated the possible relationship between SS competency and SPARS-ES. Facilities of MMS with higher SS competency scores (median 57; range 0%–77%) had higher SPARS-ES (median 3.7; range 1.6–4.0) compared to facilities of MMS with a lower SS competency score (median 24; range 17–55) having lower SPARS-ES (median 1.1; range 0.3–1.4). Since SPARS-ES could be influenced by baseline SPARS scores, we ascertained that the average baseline SPARS score for facilities with lower SPARS-ES was no different from the average baseline SPARS score of facilities with higher SPARS-ES (baseline SPARS scores 11.2 and 10.7, respectively; *p* = 0.271). Our findings thus indicate a possible relationship between quality of SS (SS competency) and SPARS impact (SPARS-ES).

### Competency by supportive supervision behavior and skill categories

SS competency scores in all categories and for all MMS was generally low, with a median score of 38% overall. The highest median category scores for all MMS was 47% achieved in the categories *educates* and *provides constructive feedback.* The categories *assures continuity* and *sets targets* had the lowest median scores of 17%. The scores for the 11 categories for the 10 MMS are provided in Additional file 2.

#### Establishes purpose

In the category *establishes purpose,* MMS had an overall median SS competency category score of 33%, with a 54 percentage point difference between MMS supervising facilities with higher and lower SPARS-ES. MMS of higher SPARS-ES facilities were good at explaining the objectives of the SPARS program and their visit purpose and reassuring staff that it was not a traditional “police and correct” intervention. None of the MMS of facilities with lower SPARS-ES related SPARS to the bigger picture during the observation visit, nor did they explain how good medicines management impacts patient care. Only one of the MMS from this group established the purpose of the visit.

#### Identifies problems

This category had an overall median of 45% and the second lowest difference in median scores between MMS grouped by their SPARS-ES, of 14% points. This suggests that most MMS have a fair understanding of how to identify problems. During the observation visit, all MMS used the indicator-based performance assessment to identify problem areas, but few prompted in-depth discussions to establish the underlying causes of the problem. Most supervisors only asked staff if they were experiencing any problems rather than probing further, which a few supervisors of higher SPARS-ES facilities did to promote fuller discussions and analysis.

#### Communicates effectively

Communication was a strength of the MMS group from high SPARS-ES facilities—three of the five MMS had very high scores, above 82%. However, the difference between the MMS from high and low SPARS-ES facilities was considerable—55 percentage points. Though overall communication skills are fairly well implemented, with a median competency score of 40%, MMS from low SPARS-ES facilities had a median score of only 27% and with only one MMS having very strong communication skills (91%).

#### Promotes participation

Most supervisors did not promote participation very well, with a median competency score of 21% and a difference between the two SPARS-ES groups of 30 percentage points, with only two MMS from facilities with high SPARS-ES reaching 65%. The supervisors rarely asked their supervisees’ opinions or prompted them to ask questions. However, the majority of supervisees did not appear to be tense or uncomfortable, and most MMS were good at listening. Although most MMS made an effort to act like one of the team at the facilities they supervised, only half managed to “fit in” successfully according to the interviews.

#### Interprets data

Interpreting data was a relative strength of both groups of supervisors, with the second highest category median score of 46%. However there was a considerable difference of 43 percentage points between the two groups. During the observation visit, some supervisors had difficulty collecting data and some rushed data collection, which might have compromised data quality. Very few supervisors encouraged staff to interpret or discuss data. However, the MMS’ understanding of and quality of the data collected was generally adequate. Data quality has been emphasized in the basic MMS training. Only a few MMS of higher SPARS-ES facilities related the performance findings to how improvements in managing medicines would improve the quality of patient care. Three of the high SPARS-ES facilities MMS scored 83% or above.

#### Solves problems

Supervisors scored an average of 36% in their ability to solve problems. A few delved into the root cause of issues adequately, but none were good at involving the supervisee and asking for their suggestions. Though MMS from high SPARS-ES facilities scored fairly high (68%), there was considerable variation in their problem solving skills (range 86%–0%). Even effective problem solvers posed the solution and asked for agreement, rather than discussing and arriving at consensus. The majority provided accurate advice and posed realistic solutions. Most MMS of lower SPARS-ES facilities did not involve the facility in-charge in the feedback session.

#### Uses tools

The ability to use SPARS tools is an important part of the MMS training, because the indicator-based performance assessment is fundamental to identifying problems and tracking progress. We found that using tools was inadequate overall (38%), with the MMS from low SPARS-ES facilities having a median score of 22% and those from high SPARS-ES facilities scoring 42%. Only one MMS used tools appropriately, getting a score of 84%. Half of the MMS updated the spider graph with new performance scores and displayed it at the facility. Generally, MMS did not use tools as an aid for motivation, but those who did supervised facilities with the highest SPARS-ES.

#### Sets targets

Setting targets was one of the most difficult tasks with an overall score of 17% and especially for the MMS of the lowest SPARS-ES facilities—four of the five MMS scored 0%. There was considerable difference between the two SPARS-ES groups—51 percentage points. Half of the MMS failed to set any targets, and because most did not follow-up on results from the previous visit, they did not check to see whether previous targets had been met. They neither set a timeline for facilities to implement changes nor provided a written record of the targets for facilities’ reference.

#### Educates

Educating the health facility staff was a relative strength, with a high median SS competency score for all MMS of 47%. The majority of supervisors attempted to identify skill gaps and educate their supervisees, although a few supervisors were completely inadequate at training (0% and 9%). In general, MMS did not take enough time to work with their supervisees to build their capacity, as data collection took priority. Training often came in the form of explanations, examples, and questions during the feedback. All health workers interviewed, however, said their supervisors were patient and clear.

#### Provides constructive feedback

Delivering accurate, *constructive feedback* was generally performed well with a median SS competency score for all MMS of 47%. However, variation was highest between the two groups in this category at 60% points. The majority of MMS did not prioritize and target their feedback but commented on all 25 indicators rather than summarizing briefly and then focusing on selected problem areas. In many facilities, the appropriate staff members were not involved in feedback; for example, the facility in-charge or other relevant staff (usually prescribers) were not present. All MMS delivered feedback in a positive way by praising health workers and not fault-finding. The highest scoring supervisors (84% and above) were excellent at involving facility staff and discussing the reasons behind issues, rather than just instituting new practices without explanation.

#### Assures continuity

This was one of the weakest SS competency categories for all supervisors, with an average score of 17%, and the lowest variation between the two groups, with just 10 percentage points. No MMS set a date for their next visit and MMS reported in interviews they would generally arrive unannounced for most of their visits. Except for one, the MMS did not discuss the previous visit, and less than half described what the supervisees needed to do by the next visit. In the interviews, supervisees and in-charges said many MMS did not regularly schedule their visit dates in advance but the majority of MMS were available in between visits if they needed assistance.

## Discussion

The quality of health services depends on the skills and performance of health workers. However, health workers are often overworked, demoralized, poorly trained, and lack sufficient recognition. This often leads to poor quality of care [12, 13]. At the same time, supervision is often seen as a nonessential resource-intense task. Central-level supervisors visit few facilities and tend to act more as fly-in–fly-out ‘police’, who do not follow up and who lack understanding of the local issues [6].

The national SPARS program has addressed several of these recognized constraints [14]. The underlying assumption of the program is that MMS who are competent in supportive supervision improve medicines management through, for example, their skills in communicating and enthusiasm to motivate staff to identify and solve problems and their inclusion of facility in-charges and district health officers in their continued efforts to effect change.

Our study shows that MMS’ supervision competency within our study group is overall low and that there is great variation between individual MMS. We acknowledge that many factors such as the supervisor, the supervision, the facility staff, the facility management, district management, work load, and experience might influence the effect of SPARS (e.g., the SPARS-ES) [11, 15]. Our findings do not support a relationship between experience of the MMS (defined as number of visits implemented) and their ability to implement SS (SS competency) or their facilities’ improvement in medicines management (SPARS-ES).

But, similar to other studies that found the importance of supportive supervision in mentoring and building capacity [5–7], our study points to a possible relationship between the MMS’ SS competency and improvement in medicines management at the facilities supervised, measured by their facilities’ SPARS-ES.

Based on our findings we suggest that SS competency should be improved through: a) focused selection of MMS, b) targeted training of MMS in specific SS behaviors and skills, and c) active management of MMS by district health officers and involvement of the in-charge.

### Focused MMS selection

Successful SPARS implementation relies on selecting MMS with the right attitude and motivation to become a supportive supervisor. Consequently, the MMS selection process will need more attention to and guidance on selection criteria than given in the past. A model MMS can be any person with training in a health-related area and does not need to be a pharmaceutically trained professional. It is more important that MMS understand their important role as supervisors in improving medicines management and assuring the community’s good health. Ability to communicate well should be a key selection criterion. MMS with high SS competency were found to have strong communication skills. Selection of MMS is crucial and DHO need better guidance in selecting MMS based on priority skills and behavior. A DHO during his interview said he “selected his MMS by default, as there was barely anyone with the right skills. There aren’t many dispensers in the district.”

Several MMS mentioned that their role made them proud, motivated, and eager to conduct SS with competency. DHOs of MMS supervising facilities that had higher SPARS-ES mentioned they wanted a results-oriented candidate with a proven track record of effectiveness and accountability. One DHO noted, “No one person is ever going to have all the skills, so if you make a team, they can support each other.” Interestingly, two MMS whose primary position was a District Assistant Drug Inspector had low SS competency. They inspected health facilities using a more traditional form of supervision by finding fault and correcting the mistakes they found. In interviews, MMS who were clinicians and drug inspectors mentioned similar objectives of SS, but the two drug inspectors seemed to find it difficult to change their practices to become more supportive mentors.

### Targeted training in specific SS behaviors and skills

The performance assessment tool using the SPARS indicators is designed to guide the MMS to identify and prioritize the problem areas to be addressed in the supervision. We found that most of the MMS were good at assessing performance by using the SPARS assessment tools, interpreting assessment data for problem identification, and providing staff education. The MMS training seems to provide acceptable skills in these competencies. However, other SS skills and behaviors need targeted training and interventions to be well implemented by MMS. Only the MMS with the highest SPARS-ES scored well on *establishing the purpose* of their visit, discussing with staff good solutions to *identified problems*, giving *constructive feedback,* and *communicating effectively*. The majority of MMS were weak in certain skills, such as *ensuring continuity* between visits, *setting targets,* and promoting staff participation. These need particular attention via focused SS training interventions.

#### Use of tools

Observation and interviews found that competent MMS use the SPARS tools to engage with facility staff. For example, during his visit, an MMS held up the spider graph he had updated and pointed to the facility’s scores, “See how good you are here in storage [paused and pointed]. If you just do the targets we’ve spoken about today, I think you’ll be at that level in prescribing next time I visit.” The supervisory book is intended to provide a written record of the feedback that facility staff can look at after the MMS has left and to provide continuity between visits, so targets are agreed and tracked. In the few facilities where the book was understood and used, it served its purpose well. However, few facilities had the supervision book on the premises. MMS said that the facility had lost it or that the MMS themselves had taken it to fill it out at home because they did not have enough time to finish it during the visit. While the supervision book was poorly implemented, the data collection tool was appropriately completed and submitted consistently, as the data collection tool was directly linked to payment of the “safari day allowance.” To increase the use of the supervisory book and ensure its availability at the facility, it is recommended that the MMS should sign the supervisory book and leave it at the facility, where it can serve to document their visit to the facility, if needed in connection with payment of the MMS’ allowance. Job aids could also be developed and implemented to ensure the purpose of tools and the visit is well explained, including the SPARS objective and strategy, within the bigger picture of health care provision.

#### Continuity and follow up

We also found that optimal and sustainable improvement requires continuity, reliable follow-up, and setting targets that are followed up. Therefore, MMS need to schedule supervisory visits consistently, so the facility has a time frame for planning and can assure that relevant staff are present to participate in the visit. The MMS also need to refer to past performance commitments or targets to check progress, including using the stock book, supervision book, and spider graph as motivators and trackers.

#### Time management

The routine SPARS performance assessments consume considerable time, especially at initial visits and at higher-level facilities. Not all MMS are able to manage their time well, which jeopardized supervision quality. Evidence suggests that poor supervisory visits are worse than no visit at all and recommend that the supervisor select one issue to address at each visit [16]. Two important recommendations emerged from our study: the MMS who will need to complete the performance assessment tool should prioritize one or two of the five SPARS areas for mentoring at each visit and schedule supervisory visits in advance with the facility. These changes should not only save time, but also avoid overwhelming the supervisee and the MMS and allow them to prepare for and focus during the visit.

### Involvement of district health officers and in-charges

Management of SPARS implementation can be better with greater involvement of the facility staff, facility in-charges, and the DHO to ensure stronger commitment, continuity, and follow-up. MMS need to play a pivotal role in ensuring management’s involvement using their effective communication skills and via debriefs, constructive feedback, and reporting both at facility and district level. MMS of the highest SPARS-ES facilities almost all provided constructive feedback involving facility staff. We found that MMS with low SS competency scores had DHOs who were either new to the post (less than six months), or according to their comments in interviews, seemed to misunderstand SPARS or appeared disgruntled with it. A previous study confirms that involving the facility and district managers strengthened commitment to the process and expedited change [16]. An understanding of SPARS and the roles and responsibilities of managers (MMS, DHOs, regional pharmacists, and facility management) within SPARS must be very clear. A process to orient new managers to SPARS should be put in place within their first month of arrival. The facility in-charge plays a crucial role in directing work priorities and garnering support from their staff. Some facility in-charges were not present during the feedback sessions. Three in-charges mentioned lack of continuity as their biggest complaint with SPARS*:* “*He comes when the other programs are here. They have booked, so we don’t have enough hours for him too.”* It is crucial that the MMS plan their visit well in advance and in conjunction with the facility staff, including the in-charge, to ensure their availability and involvement in the debriefing process.

### Limitations

This study has important limitations. Supervisors and health workers were asked to conduct their supervision visits as they normally would. To encourage honesty, we assured interviewees that we would keep their identities confidential and that their comments would not have any professional or personal impact. However, because being observed affects people’s behavior, the study may not have captured typical interactions, and privacy concerns may have prevented people from sharing their honest opinions.

Our sample only included 10 MMS, all men, and we cannot assume that results reflect competency of all MMS, especially because 19% of the MMS in the country are women. However, the range of skills, educational backgrounds, and professions in the studied group was wide. In addition, we only observed one supervisory visit for each MMS. However, the results produce coherent findings. Arguably, observing only one visit per MMS should suffice, as SPARS implementation had become a routine practice for the experienced MMS, and implementation would differ only slightly for the same MMS at different visits over time because each MMS has the same competencies, skills, and behaviors.

A maximum of up to five supervision visits to a facility was included in measuring SPARS-ES. We limited the measure to five visits to standardize the SPARS-ES score and excluded visits where the effect curve would be flattened, which would have misrepresented facilities’ SPARS improvements. The baseline SPARS score of the facilities with higher versus lower SPARS-ES did not differ significantly, that is, the difference in SPARS-ES is not likely to be linked to differences in initial SPARS score or starting point on the SPARS improvement curve.

The SS competency assessment tool needs validation and reliability testing. The fact that MMS supervising facilities with higher SPARS-ES had higher SS competency scores than those supervising facilities with lower SPARS-ES suggests construct validity, and the comments of interviewees suggest face validity of the competency measurement tool. Some of the observational ratings were more subjective, which could lead to ratings differing by raters. However, the same raters assessed competency of each MMS while unaware of facility SPARS-ES, which was intended to ensure the same subjective assessment.

Lastly, the small sample size precludes statistical analysis of the relationship between MMS SS competency scores, MMS experience (number of visits), and facility SPARS-ES.

## Conclusions

This study aimed to assess the competency in SS of MMS in Uganda, evaluating 11 categories of desired SS behaviors and skills. SS competency of the 10 observed MMS was poor, with a median score of just 38%. The study’s limitations notwithstanding, the results suggest that MMS SS competency is positively related to SPARS effectiveness scores of the facilities they supervise.

We found that all MMS were good at using performance assessment in their supervision involving use of assessment tools, assessing and interpreting data, problem identification, and fair in staff education; the MMS with the highest facility SPARS-ES were better at establishing the purpose of their visit, discussing solutions to identified problems with staff, educating staff, and giving constructive feedback. All MMS were found to be weak in ensuring continuity between visits.

We recommend strategies to improve SS competency. Enhancement strategies should focus on MMS selection, emphasizing attitude, communication skills, and motivation. Targeted MMS training in supportive supervision should be conducted, focusing on the weakest of the 11 categories that are important for supportive supervision. Better involvement of the district health officers, health facility staff, and in-charges in the management of medicines and implementation of SPARS should also be promoted.

## Additional files

Additional file 1: Interview questionnaire. (DOCX 54 kb)

Additional file 2: Supportive supervision categories scores for the observed supervisors. (DOCX 18 kb)

## Abbreviations

DHO: District health officers
MMS: Medicines management supervisors
SPARS: Supervision, Performance Assessment, Recognition Strategy
SPARS-ES: SPARS effectiveness scores
SS: Supportive supervision
