# Supplementary material for: Competency in supportive supervision: a study of public sector medicines management supervisors in Uganda
Source: J Pharm Policy Pract. 2017 Oct 11;10:33. doi: 10.1186/s40545-017-0121-y (PMC5637320; doi:10.1186/s40545-017-0121-y)
Supplement: Supplementary file 1 — Interview questionnaire. (DOCX 54 kb) [file 40545_2017_121_MOESM1_ESM.docx]

# Additional File 1: Interview questionnaire

Questions about the MMS supervising the health facility

| How would you describe the MMS’ work methods?  □ Hands on – the MMS helps you with improving the standards in the health facility. For example, the MMS helps you fill out stock cards, labelling the shelves etc.  □ Educational – the MMS explains to you how to improve the standard of the health facility. For example, the MMS will tell you how to calculate AMC but will not sit down and help you calculate  □ Policing – the MMS will tell you what has to be improved but will not tell you how to improve  □ Other – describe the work method  ________________________________________________________________________________________________________________________________________________________________________________________________________________________________________________________________________________________________________________________________________ | |
| --- | --- |
| Who does the MMS mentor when he comes for support supervision (tick all that is appropriate)?  □ Health facility in-charge  □ Pharmacy in-charge  □ Store in-charge  □ Other _______________________________  □ All the health facility staff | |
| What do you think of the supervision that you receive?  □ I like the supervision □ I don’t like the supervision  □ The MMS is very helpful □ The supervision takes too much time  □ I think the MMS helps us improve the standard of the health facility  □ I think the MMS’ work results in a better service to the patients  □ I think the MMS’ work results in better availability of medicine and health supplies  □ Other _________________________________________________________  □ Other _________________________________________________________ | |
| How would you describe the management of the health facility?  □ The management encourages me to improve on the areas identified by the MMS  □ The management does not want me to use time on improving in the areas identified by the MMS  □ Other ______________________________________________  □ Other ______________________________________________  □ Other ______________________________________________ | How many times have the DHO visited your health facility in the last year?  □ 0  □ 1time  □ 2 times  □ > 2 times |
