# Supplementary material for: Competency in supportive supervision: a study of public sector medicines management supervisors in Uganda
Source: J Pharm Policy Pract. 2017 Oct 11;10:33. doi: 10.1186/s40545-017-0121-y (PMC5637320; doi:10.1186/s40545-017-0121-y)
Supplement: Supplementary file 2 — Supportive supervision categories scores for the observed supervisors. (DOCX 18 kb) [file 40545_2017_121_MOESM2_ESM.docx]

**Additional File 2: Supportive supervision categories scores for the observed supervisors**
